# Supplementary material for: An open-source framework for large-scale, flexible evaluation of biomedical text mining systems
Source: J Biomed Discov Collab. 2008 Jan 29;3:1. doi: 10.1186/1747-5333-3-1 (PMC2276192; doi:10.1186/1747-5333-3-1)
Supplement: Additional file 1 — Evaluation platform source code. The additional file contains the software evaluation framework discussed in this paper. The most current release can be downloaded from SourceForge.net [1]. See the accompanying README file for instructions on installation and use. Also included in the distribution is a collection of UIMA wrappers for some commonly used BioNLP tools and annotated corpora. [file 1747-5333-3-1-S1.zip › bionlp_uima_repository-1.0.1/docs/api/edu/uchsc/ccp/uima/cc/summary/opendmap/TestPrinter.html]

TestPrinter (Center for Computational Pharmacology Util/UIMA API)


|  |  |  |
| --- | --- | --- |
| **Overview** \| **Package** \| **Class** \| **Tree** \| **Deprecated** \| **Index** \| **Help** | |  |
| PREV CLASS   NEXT CLASS | **FRAMES**    **NO FRAMES**     **All Classes** |  |
| SUMMARY: NESTED \| FIELD \| CONSTR \| METHOD | DETAIL: FIELD \| CONSTR \| METHOD |  |


---


## edu.uchsc.ccp.uima.cc.summary.opendmap Class TestPrinter

```
java.lang.Object
  org.apache.uima.resource.Resource_ImplBase
      org.apache.uima.resource.ConfigurableResource_ImplBase
          org.apache.uima.collection.CasConsumer_ImplBase
              edu.uchsc.ccp.uima.cc.summary.opendmap.TestPrinter
```

**All Implemented Interfaces:**: org.apache.uima.collection.base\_cpm.CasObjectProcessor, org.apache.uima.collection.base\_cpm.CasProcessor, org.apache.uima.collection.CasConsumer, org.apache.uima.resource.ConfigurableResource, org.apache.uima.resource.Resource

---

``` public class TestPrinter extends org.apache.uima.collection.CasConsumer_ImplBase implements org.apache.uima.collection.base_cpm.CasObjectProcessor ```

This class looks through the JCas for annotations of a certain type
and prints them in a serialized format appropriate for later comparison
using the TestChecker.

This code is used for automated testing. The annotations
are printed in a compact serialized fashion.

The parameters used are:

- OutputToFile: Set to tru if the output should be written to a file.
  By default output is written to standard output.- OutputFileName: The name for the output file if one is used.- OutputMentionTypes: A string of the form type,slot,slot,...;type,slot,slot, ... describing
      the mention types and slots to include in the serialized output string.- FormatForWiki: Set to true if the output should be generated in
        a format that enables it to be cut and pasted in the UCHSC wiki.

**Author:**
:   R. James Firby

---

**Field Summary**

**Fields inherited from interface org.apache.uima.resource.Resource**

`PARAM_AGGREGATE_SOFA_MAPPINGS, PARAM_CONFIG_PARAM_SETTINGS, PARAM_PERFORMANCE_TUNING_SETTINGS, PARAM_RESOURCE_MANAGER, PARAM_UIMA_CONTEXT`


**Constructor Summary**

`TestPrinter()`


| **Method Summary** | |
| --- | --- |
| `void` | `initialize()`             The standard UIMA initialize method for this processor. |
| `void` | `processCas(org.apache.uima.cas.CAS cas)`             The standard UIMA process method. |

**Methods inherited from class org.apache.uima.collection.CasConsumer\_ImplBase**

`batchProcessComplete, collectionProcessComplete, destroy, getProcessingResourceMetaData, initialize, isReadOnly, isStateless, processCas, reconfigure, typeSystemInit`

**Methods inherited from class org.apache.uima.resource.ConfigurableResource\_ImplBase**

`getConfigParameterValue, getConfigParameterValue, setConfigParameterValue, setConfigParameterValue`

**Methods inherited from class org.apache.uima.resource.Resource\_ImplBase**

`getCasManager, getLogger, getMetaData, getResourceManager, getUimaContext, getUimaContextAdmin, setLogger, setMetaData`

**Methods inherited from class java.lang.Object**

`clone, equals, finalize, getClass, hashCode, notify, notifyAll, toString, wait, wait, wait`

**Methods inherited from interface org.apache.uima.collection.base\_cpm.CasObjectProcessor**

`processCas, typeSystemInit`

**Methods inherited from interface org.apache.uima.collection.base\_cpm.CasProcessor**

`batchProcessComplete, collectionProcessComplete, getProcessingResourceMetaData, isReadOnly, isStateless`

**Methods inherited from interface org.apache.uima.resource.ConfigurableResource**

`getConfigParameterValue, getConfigParameterValue, setConfigParameterValue, setConfigParameterValue`

**Methods inherited from interface org.apache.uima.resource.Resource**

`getLogger, getMetaData, getResourceManager, getUimaContext, getUimaContextAdmin, setLogger`

**Constructor Detail**

### TestPrinter

```
public TestPrinter()
```


**Method Detail**

### initialize

```
public void initialize()
                throws org.apache.uima.resource.ResourceInitializationException
```

:   The standard UIMA initialize method for this processor. Get the parameter
    values and initialize the summarizer.

    :   **Overrides:**: `initialize` in class `org.apache.uima.collection.CasConsumer_ImplBase`
    :   **Throws:**: `org.apache.uima.resource.ResourceInitializationException`

---


### processCas

```
public void processCas(org.apache.uima.cas.CAS cas)
                throws org.apache.uima.resource.ResourceProcessException
```

:   The standard UIMA process method. Find the desired mentions in the JCas and
    print them out as serialized strings.

    :   **Specified by:**: `processCas` in interface `org.apache.uima.collection.base_cpm.CasObjectProcessor`
    :   **Throws:**: `org.apache.uima.resource.ResourceProcessException`


---


|  |  |  |
| --- | --- | --- |
| **Overview** \| **Package** \| **Class** \| **Tree** \| **Deprecated** \| **Index** \| **Help** | |  |
| PREV CLASS   NEXT CLASS | **FRAMES**    **NO FRAMES**     **All Classes** |  |
| SUMMARY: NESTED \| FIELD \| CONSTR \| METHOD | DETAIL: FIELD \| CONSTR \| METHOD |  |


---
